# Dendrimer Anion-Exchange Stationary Phase for Separation of Oligonucleotides

**DOI:** 10.3390/molecules27051491

**Published:** 2022-02-23

**Authors:** Sylwia Studzińska, Szymon Bocian, Anna Kilanowska, Bogusław Buszewski

**Affiliations:** Chair of Environmental Chemistry and Bioanalytics, Faculty of Chemistry, Nicolaus Copernicus University in Toruń, 7 Gagarin Str., 87-100 Toruń, Poland; bocian@chem.umk.pl (S.B.); ania.kaczmarkiewicz@gmail.com (A.K.); bbusz@chem.umk.pl (B.B.)

**Keywords:** oligonucleotide, dendrimer stationary phase, ion-exchange chromatography, separation, interaction

## Abstract

Oligonucleotides are used in many research areas. Thus, there is a need for their successful separation methods. Ion-exchange chromatography is the most popular separation technique, but it has limitations for these compounds. For this reason, new stationary phases are developed in order to increase separation selectivity. This study aimed to apply a series of dendrimer anion exchangers with various bonded layers to separate oligonucleotides by using different mobile phases to find conditions that allow sufficient separation. The number of anion-exchange layers, type of salt, and pH significantly impacted the oligonucleotide analysis. The developed chromatographic method was characterized by adequate selectivity for oligonucleotides differing in sequence length. It is essential to underline that the number of bonded layers appeared to have a significant influence, and the three layers appeared optimal. Based on our results, it may be concluded that the dendrimer stationary phases can be successfully used as an alternative to commonly applied packing materials in ion-exchange chromatography.

## 1. Introduction

Oligonucleotides are compounds built of many nitrogen bases, sugars (ribose or deoxyribose), and phosphate groups [1]. Their structure consists of nucleotides bounded by a phosphodiester bond in a single strand. Consequently, oligonucleotides are polar, negatively charged compounds with multiple charges derived from phosphate groups [2]. Through inter-molecular self-complementarity structures or intra-molecular structures, they may form hairpin loops. Oligonucleotides are used in many research areas, including as primers in polymerase chain reactions, as probes for DNA sequencing, for the characterization and tracking of nucleic acids in biological systems, and as therapeutics in antisense and gene therapy [1,2,3,4].

It is necessary to study oligonucleotide analysis, especially in impurity determination, degradation, or biotransformation product analysis [5]. Separation techniques are mainly used to separate and determine oligonucleotides [5,6]. The two most popular analytical tools are ion-pair liquid chromatography and ion-exchange chromatography [6,7,8]. In the first technique, the amines are used as mobile phase additives. This increases the selectivity but, in some cases, reduces the sensitivity [6]. Similar separation selectivity may be obtained in ion-exchange chromatography, where the retention is based on electrostatic interactions between the negatively charged oligonucleotide and positively charged stationary phase surface [7,8,9,10,11]. The utilization of anion-exchange chromatography allows an elevated temperature or pH to be employed to control oligonucleotides’ separation selectivity by producing fully or partially denaturing conditions [8]. Adsorbents with quaternary ammonium ligands are widely used as stationary phases. Different lengths of alkyl chains can be bounded to the quaternary nitrogen atom—usually short ones such as trimethylamine or diethylmethylamine [8,9,10,11]. They were successfully applied to separate unmodified, single-stranded OGNs and modified oligonucleotides but some disadvantages were noticed—for example, a lengthy analysis time or insufficient separation [9,10,11]. Therefore, chromatographic methods of oligonucleotide analysis need improvements, such as proper, careful mobile phase composition changes or the synthesis of new and alternative stationary phases and their application.

Dendrimer stationary phases are a unique separation material for ion-exchange chromatography. Such materials may be synthesized on the polymer support or inorganic materials, e.g., silica gel [12,13]. Silica gel ensures rapid mass transfer, good loadability, and high reproducibility. Unfortunately, silica reveals limited stability in the pH range 2–10 [12,13]. One of the newest methods of stationary phase preparation with quaternary ammonium groups is the methodology introduced by Pohl and his co-workers [14,15]. A hyperbranched anion-exchange polymer stationary phase is created during the synthesis, containing quaternary ammonium, tertiary, and secondary amine groups. Quaternary ammonium groups possess a positive charge in the pH range 1–14 [14,15,16,17,18]. Thus, they are strong anion exchangers. On the other hand, tertiary, secondary, and primary amino groups can be positively charged below pH around 9. As a result, the ion-exchange capacity of the dendrimer anion exchanger may be controlled during the synthesis and analysis by changing the pH of the mobile phase [14,15,16,17,18].

Dendrimer anion exchangers may be applied to separate inorganic anions and nucleotides [16,17,18,19,20]. Considering the properties of dendrimer anion exchangers described in the literature, it seems reasonable to use them to separate oligonucleotides. Hence, the goal of this study was to apply a series of dendrimer anion exchangers with a different number of bonded layers for the separation of oligonucleotides using various mobile phases. This study aimed to determine how useful dendrimeric stationary phases will be in analyzing oligonucleotides.

## 2. Results and Discussion

### 2.1. Stationary Phase Characterization

**Figure 1:**
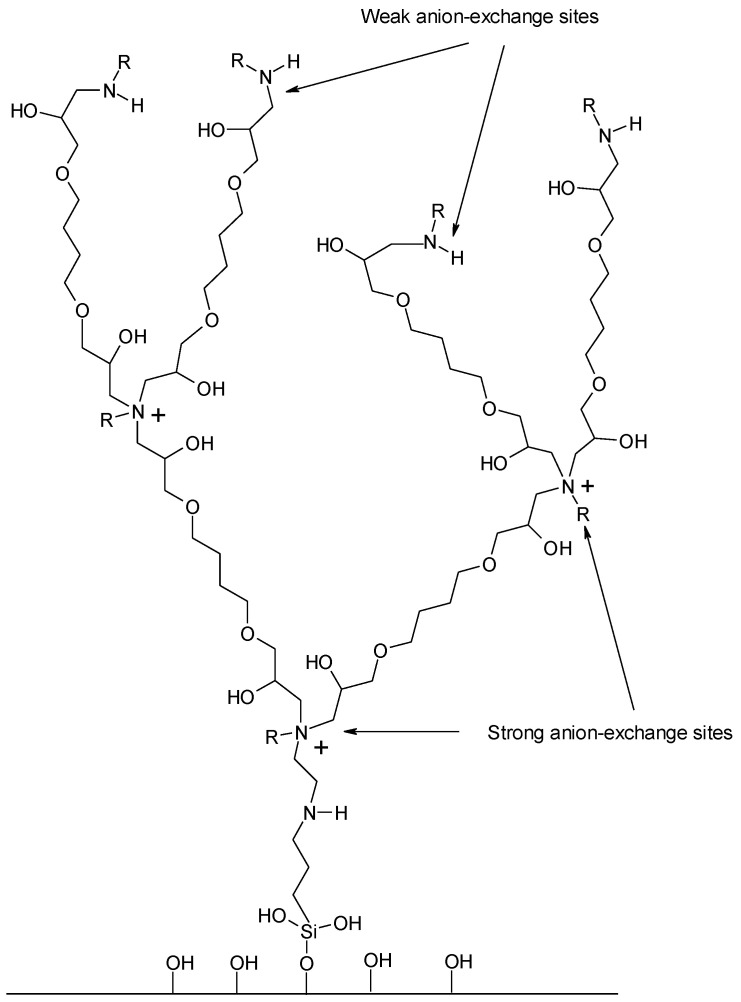
Schematic structure of dendrimer anion exchanger obtained after two reaction cycles.

In this study, four different chromatographic stationary phases were applied. These materials contained one, two, three, and four bonded anion exchange layers. The exemplary structure is presented in Figure 1 and the elemental composition of the dendrimer-bonded materials is shown in Table 1.

It is essential to underline that the carbon, nitrogen, and hydrogen content increases with the reaction cycles. However, the increase is lower, as expected from the dendrimer structure. This confirms that the dendrimer structure is not ideal, and not all nitrogen atoms react to obtain quaternary ammonium salts. It results from steric hindrance during the further steps of modification. Each reaction step makes the structure of ligands denser, which hampers the reaction and penetration of analytes into ligands during the separation procedure [16,17,18].

### 2.2. The Influence of the Salt in the Mobile Phase on Oligonucleotide Retention

The sequences and molecular masses of the oligonucleotides tested during the study are presented in Table 2.

The impact of salt type in the mobile phase on the retention of the tested oligonucleotides was studied. Two inorganic salt solutions, as well as two organic salts, were selected. The results obtained for one of the stationary phases are presented in Table 3.

The highest *k* values were determined for ammonium acetate and ammonium formate. This effect is related to the lower elution strength of organic salts in ion-exchange chromatography, resulting from their lower affinity to the anion-exchange site. A significant peak asymmetry was noticed for both salts ranging between 1.5 and 1.8. In consequence, ammonium formate and acetate were excluded from further studies. The greatest elution strength was observed for sodium perchlorate (Table 3). Unfortunately, it provides poor peak shapes of oligonucleotides. Based on the retention factors and peak symmetry, sodium chloride was chosen as a mobile phase component.

### 2.3. The Impact of the Length, Sequence, and Modification of Oligonucleotides on Their Retention on Dendrimer Stationary Phases

Another effect observed during the study was related to the oligonucleotides’ sequence and length. An increase in *k* values was noticed together with the sequence length (Table 2 and Table 3). However, in some cases, the difference between *k* values was not significant, despite differences in sequence length, e.g., OL5 and OL6 for NaClO_4_ and OL4 and OL5 for HCOONH_4_. This was probably a consequence of the observed peak asymmetry, influencing the integration and retention time determination. Other trends were observed when using inorganic salts as mobile phases. These were related to the higher strength of electrostatic interactions with the stationary phase surface. As the number of nucleotides building the oligonucleotide increases, negatively charged phosphate groups also increase. Therefore, the resultant negative charge of the oligonucleotide increases simultaneously, which causes greater interaction with the positively charged quaternary ammonium groups at the stationary phase surface. This effect appears to be sequence-independent (e.g., for OL1 and OL4, for OL2 and OL5, OL3, and OL6).

In most cases, the *k* values are very similar (Table 3). Therefore, it may be concluded that the main impact on the retention of oligonucleotides on an anion exchanger is the electrostatic interactions, consistent with the theory of ion chromatography. It also shows that dendrimer stationary phases can be used to separate oligonucleotides differing in length (contaminants such as shortmers and longmers, or simple metabolites). Unfortunately, our study did not prove that dendrimeric stationary phases allow the separation of oligonucleotides differing by only one nucleotide in the sequence, e.g., 20-mer and 19-mer. Moreover, they cannot be used for studies of sequential isomers or isobaric metabolites because the structural selectivity is neglectable.

Oligonucleotides whose sequences were modified within each phosphate group (PS) or each ribose (ME and MOE) were also used in this study (see Table 2). They had sequences analogous to oligonucleotide OL4 (Table 2). Therefore, it was possible to compare the effect of modification type on the retention of the oligonucleotides analyzed using dendrimer stationary phases in ion-exchange chromatography mode. Based on the data summarized in Table 3, it can be concluded that, for CH_3_COONH_4_ and HCOONH_4_, the *k* values were comparable for OL4, PS1, ME, and MOE. This indicated that, for organic salts, the modification of the oligonucleotide has no effect on its retention. If the oligonucleotides are of the same length, the *k* values are similar. The unmodified OL4 had similar retention to the 2′-O-methoxyethyl-modified oligonucleotide MOE. Higher *k* values were observed for the oligonucleotide with 2′-O-methyl modification in the ribose moiety (ME) (Table 3). Such an effect probably indicates that hydrophobic interactions also impacted the oligonucleotides’ retention at the dendrimer stationary phase. Using inorganic salts in the mobile phases, we can distinguish oligonucleotides with the same sequence and length but different types of modification, but this applies only to 2′-O-methyl modification.

### 2.4. The Influence of Mobile Phase pH on Oligonucleotide Retention at the Dendrimer Stationary Phase

**Figure 2:**
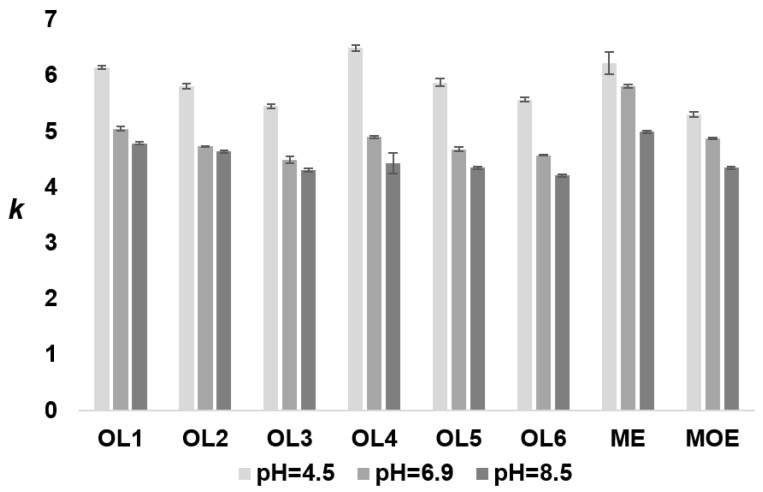
The impact of sodium chloride pH on the oligonucleotides’ retention. Experimental conditions: stationary phase with III dendrimer layers; gradient elution program: 0 min—0.125 M NaCl, 5 min—0.4 M NaCl, 15 min—0.4 M NaCl; flow rate 0.3 mL/min; autosampler and column temperature: 35 °C; injection volume: 1 µL; detection UV at λ = 260 nm.

The effect of sodium chloride solution (pH 4.5, 6.9, and 8.5) on oligonucleotide retention was tested. The results show that as the pH value of the salt increased, the retention rates of all tested oligonucleotides decreased (Figure 2).

Such trends were observed regardless of the oligonucleotide length and type of modification. Apart from quaternary ammonium groups, whose charge is pH-independent, secondary and tertiary amines, with a positive charge at a lower mobile phase pH, are also present in the stationary phase structure. The synthesis of dendrimer stationary phases is a multi-step process. Firstly, the surface of the silica gel is modified with propylamine groups and next with dendrimer groups. However, not all available aminopropyl groups are blocked during further synthesis steps; some will interact with the analyte. These weak ion exchangers remain open for the oligonucleotides during the chromatographic process.

Consequently, higher *k* values at pH = 4.5 were determined (compared to higher pH) due to the more significant number of protonated anion-exchange groups on the surface of the stationary phase (Figure 2). Nevertheless, it should be emphasized that as the pH value of the NaCl solution decreased, the oligonucleotide peak shapes became asymmetric (f_AS_ greater than 1.5). This effect was probably due to greater retention and interaction between the dendrimer surface and oligonucleotide. For this reason, a pH of 6.9 was selected for further studies.

In contrast, at pH = 8.5, a higher concentration of hydroxide ions is present in the solution. In ion chromatography, hydroxide ions have significant elution strength on anion exchangers. Thus, the increasing hydroxide anion concentration results in a decrease in retention compared to neutral NaCl solution. Additionally, it is necessary to emphasize that the pKa of secondary amine in the structure of bonded ligands is around 8. This confirms that a significant decrease in protonation in the pH range 6.9–8.5 was observed, and thus a reduction in the oligonucleotides’ retention.

### 2.5. The Impact of Dendrimer Layers on the Oligonucleotide Retention

**Figure 3:**
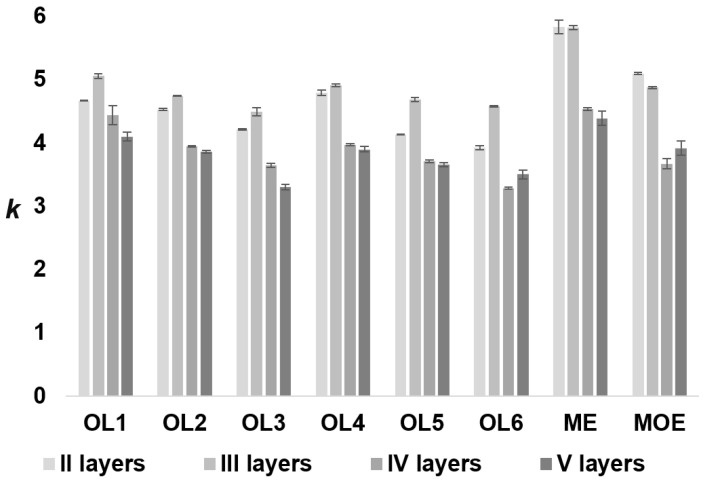
The influence of anion-exchange layers in the dendrimer structure of the stationary phases on the retention of oligonucleotides. Experimental conditions: gradient elution program: 0 min—0.125 M NaCl, 5 min—0.4 M NaCl, 15 min—0.4 M NaCl; pH = 6.9; flow rate 0.3 mL/min; autosampler and column temperature: 35 °C; injection volume: 1 µL; detection UV at λ = 260 nm.

The last stage of oligonucleotide retention studies using dendrimer stationary phases was studying the effect of the number of anion-exchange layers on the retention of oligonucleotides, measured as *k* values. The results are presented in Figure 3.

An increase in retention was initially observed with an increasing number of anion-exchange layers in the dendrimer structure of the stationary phase (Figure 3) for unmodified oligonucleotides (OL1–OL6), with the exception of ME and MOE. This trend concerns II and III layers of anion exchangers and is related to the greater number of NR_3_^+^ groups on the surface of the stationary phase with three anion-exchange layers. The electrostatic interactions between this material and the oligonucleotides were more effective than the stationary phase with two anion-exchange layers. A further increase in dendrimer layers to four resulted in lower oligonucleotide retention (Figure 3). This results from the steric barrier formed due to greater ligand branching and the denser structure [16,17,18,19,20]. The decrease in *k* for the stationary phase with IV and V anion-exchange layers is due to the lower mass transfer of high-molecular-weight biomolecules in the dendrimer layers. Moreover, the 100 Å pores at the silica surface are probably significantly obstructed when dendrimers with IV and V layers are synthesized. The observed effect is perhaps a consequence of two phenomena. Firstly, the penetration of oligonucleotides that are giant molecules between dendrimer branches to anion-exchange sites is hampered. On the other hand, excluding oligonucleotides from the pores may also play a significant role for IV and V layers. It results in a decrease in retention. It is essential to underline that the highest decrease is observed between the III and IV bonded layers.

Further reaction cycles do not significantly reduce the retention. However, the exception was OL6 and MOE, for which the *k* values increased slightly between layers IV and V, indicating that perhaps the steric barrier effect is not critical for all tested oligonucleotides. Therefore, it is the general trend for macromolecular oligonucleotides and dendrimeric stationary phases. Summarizing, in the case of stationary phases with two and three layers, oligonucleotides penetrate branched ligands and interact with quaternary ammonium groups. This becomes difficult when the silica gel is modified with more anion-exchange layers. Therefore, it may be assumed that oligonucleotide retention on dendrimer anion exchangers is determined by two factors: electrostatic interactions with NR_3_^+^ groups (main effect) and the ability to penetrate stationary phase layers (supplementary effect).

### 2.6. Oligonucleotides’ Mixture Separation Using Dendrimer Stationary Phases in Ion-Exchange Chromatography

**Figure 4:**
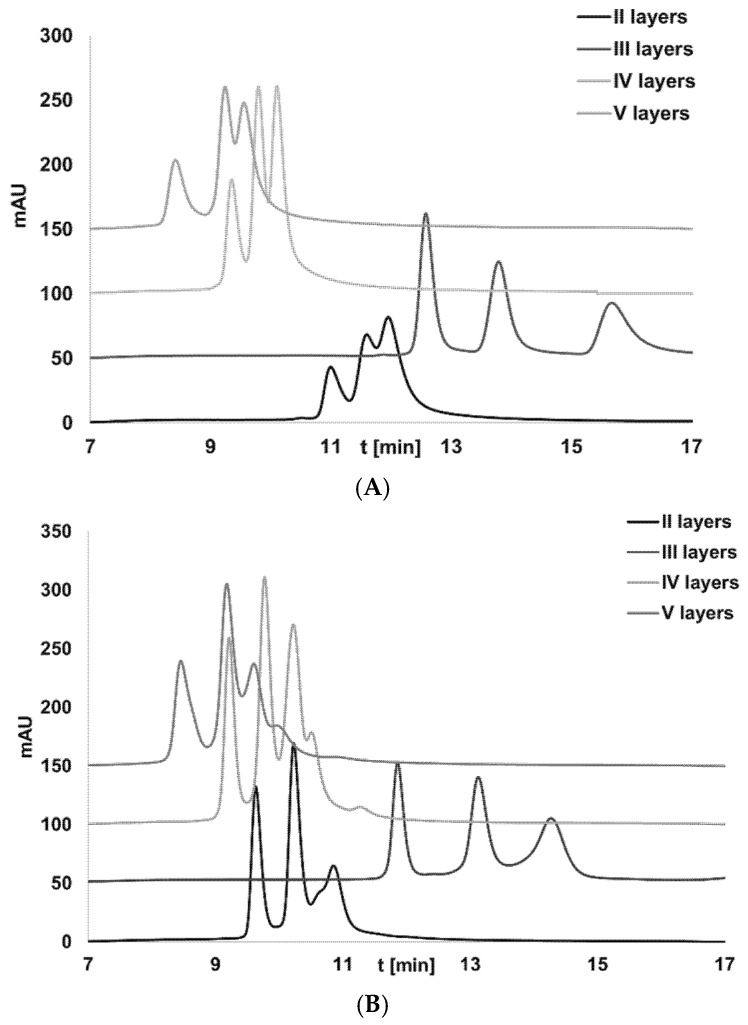
Separation of oligonucleotide mixtures using dendrimer anion exchangers: (**A**) mixture of OL1, OL2, OL3; (**B**) mixture of OL4, OL5, OL6. Experimental conditions: gradient elution program 0 min—0.125 M NaCl, 5 min—0.4 M NaCl, 15 min—0.4 M NaCl; pH 6.9; flow rate 0.3 mL/min; column and autosampler temperature 35 °C; injection volume: 2 µL; UV detection λ = 260 nm; elution order for (**A**) OL3, OL2, OL1; for (**B**) OL6, OL5, OL4.

An attempt to separate two oligonucleotide mixtures (OL1, OL2, OL3, and OL4, OL5, OL6) was made for all tested stationary phases. The mobile phase was the same in the case of all tested mixtures. The decrease in OGN retention was noticed for dendrimers with IV and V layers because of the effects described in the previous section. Although greater retention was determined for V layers, the separation selectivity (especially between 10-mer and 15-mer) was greater for the anion exchanger with IV layers (Figure 4). This is a consequence of the steric hindrance between the oligonucleotide and stationary phase with branched five layers. Both stationary phases will not provide satisfactory results for oligonucleotide mixture separation. A similar conclusion may be drawn for the stationary phase with II anion-exchange layers. Complete separation of both mixtures was obtained for the adsorbent with III anion-exchange layers (Figure 4). It is likely that two effects play an essential role: a sufficient number of NR_3_^+^ groups (allowing for effective electrostatic interactions) and low steric hindrance (allowing oligonucleotides to penetrate stationary phase pores). Our results indicate that sodium chloride and a dendrimer stationary phase with three layers are preferred for oligonucleotide separation.

The separation results are similar when comparing the two mixtures because of the samples’ similar lengths of three oligonucleotides (10-mer, 15-mer, 20-mer) (Figure 4). As mentioned earlier, the retention of oligonucleotides on the dendrimer stationary phases in ion-exchange chromatography is based on the difference in their length (the net charge of the molecule is changing simultaneously).

To summarize, the retention data reproducibility was good (relative standard deviations were lower than 15%). The developed separation method determined the intraday and interday precision values (by analyzing oligonucleotide mixtures at two different concentration levels). The intraday precision was lower than 3.4–3.9% for low and 2.8–4.3% for high concentrations. The interday precision values were in the range of 3.5–4.8%. The lifetime of the chromatographic column with III dendrimer layers was used for two months of daily operation (4 days per week for around 6 h per day) during the study. After this time, a slight tailing of peaks was observed, suggesting a slow loss of efficiency.

## 3. Materials and Methods

### 3.1. Materials

The anion exchangers were prepared by modifying silica gel Kromasil 100 (Akzo Nobel, Bohus, Sweden) with particle size 5 μm and an average pore diameter of 100 Å. The specific surface area of bare silica gel was 313 m^2^/g, and pore volume was 0.87 cm^3^/g. The following reagents were used for the chemical modification of the silica gel support material: methylamine (MA40% in water, *v*/*v*) and 1,4-butanedioldiglycidylether (BDDE 95% in water, *v*/*v*), both purchased from Sigma-Aldrich Chemie (Steinheim, Germany).

The detailed synthesis procedure is described in the previous study [16,17,18]. Each reaction cycle provides the deposition of the following layer of the anion exchanger. Thus, the number of reaction cycles corresponds to dendrimer generation and bonded layers of anion exchangers.

In the previous studies, dendrimer ion-exchange stationary phases were stable in the pH range 3.0–9.0. The application of material at higher pH strongly reduces the column lifetime. During laboratory work, the reproducibility of the stationary phases at various reaction steps allows us to obtain an RSD lower than 10%.

During the study, unmodified and modified oligonucleotides differing in modification types and length were applied. Both unmodified and phosphorothioate were purchased from Sigma-Aldrich (Gillingham, Dorset, UK), while oligonucleotides with 2′-O-methyl, 2′-O-methoxyethyl modifications were obtained from Eurogentec (Seraing, Liege, Belgium). Table 2 presents the sequences, molecular masses, and types of modifications of all studied compounds. The lyophilized solutes were dissolved in deionized water to the concentration of 100 µM, and next, they were diluted to a proper concentration.

Mobile phases were prepared using the following solvents: deionized water (Milli-Q system, Millipore, El Passo, TX, USA), and high-purity salts such as ammonium formate and ammonium acetate, sodium chloride, sodium perchlorate (Sigma-Aldrich, Gillingham, Dorset, UK). Salts were prepared by dissolving an appropriate amount in deionized water and adjusting the pH using sodium hydroxide (Merck KGaA, Darmstadt, Germany) or hydrochloric acid (Merck KGaA, Darmstadt, Germany). Before use, mobile phases were filtered through 0.2 µm nylon filters (Supleco Analytical, Bellefonte, PA, USA).

### 3.2. Apparatus and Chromatographic Conditions

During the study, a liquid chromatograph from Shimadzu Prominence (Kioto, Japan), equipped with a quaternary pump, degasser, autosampler, column thermostat, and spectrophotometric diode-array UV-Vis detector (DAD), was used. The data were collected using LabSolution version 5.8 with a computer data acquisition station.

The oligonucleotide retention studies were performed using the following gradient elution program: 0 min—0.125 M of salt, 5 min—0.4 M of salt, 15 min—0.4 M of salt. Four mobile phases were used to elute the oligonucleotides, namely sodium chloride, sodium perchlorate, ammonium formate, and ammonium acetate. Salt concentrations were in the range of 0.1–0.4 M. Fresh salt solutions were prepared daily. For these salts, the impact of three different pH values (4.5, 6.9, 8.5) on the retention of oligonucleotides was tested. The pH was modified by the addition of HCl and NaOH, respectively. The flow rate was 0.3 mL·min^−1^, while the temperature of the autosampler and column equaled 35 °C. The UV detection wavelength was selected as λ = 260 nm, while the injection volume was 1 µL of 25 µM solutions.

The material under study was packed into 150 mm × 2.1 mm PEEK columns. All columns were packed using a DT122 packing pump (Haskel, Burbank, CA, USA) under the pressure of 25 MPa.

## 4. Conclusions

One of the most critical parameters in analyzing oligonucleotides by liquid chromatography is the resolution. This is a consequence of peak symmetry and, what follows, the efficiency and selectivity of chromatographic columns. The results obtained for oligonucleotides often require changes and improvements of the chromatographic system. The most rapidly developing direction of research on oligonucleotides using liquid chromatography is the search for new stationary phases for their analysis. For the first time, in the present study, dendrimer stationary phases were used to analyze oligonucleotides. The presence of quaternary ammonium groups in their structures allowed for electrostatic interactions between them and negatively charged phosphate groups in the oligonucleotides’ structures. Therefore, dendrimer stationary phases can be successfully used as an alternative to commonly applied packing materials in ion-exchange chromatography. The developed chromatographic method is characterized by adequate selectivity for oligonucleotides with differing sequence lengths.

Nevertheless, high inorganic salt concentrations in the mobile phase limit the applicability of mass spectrometry detection, while UV-Vis detection has lower sensitivity. For these reasons, the dendrimer stationary phase with three layers may be utilized to analyze oligonucleotide impurities formed during their synthesis. In this case, their concentration is high, and consequently, the method’s high sensitivity is not required.

## Figures and Tables

**Table 1:** Elemental composition of prepared series of anion exchangers.

Number of Reaction Cycles	Nitrogen Content (%)	Carbon Content (%)	Hydrogen Content (%)
1	1.72	10.63	1.87
2	1.78	12.67	2.19
3	1.82	14.42	2.45
4	1.88	16.45	2.80

**Table 2:** Sequences and molecular masses of oligonucleotides used in the present study.

Shortcut	Sequence (5′→3′)	Modification	Number of Nucleotides	Molecular Mass (Da)
OL1	GCCCAAGCTGGCATCCGTCA	-	20	6063
OL2	GCCCAAGCTGGCATC	-	15	4538
OL3	GCCCAAGCTG	-	10	3013
OL4	GCTAGCTAGCTAGCTAGCTA	-	20	6117
OL5	GCTAGCTAGCTAGCT	-	15	4568
OL6	GCTAGCTAGC	-	10	3028
PS	GCTAGCTAGCTAGCTAGCTA	phosphorothioate	20	6368
ME	GCTAGCTAGCTAGCTAGCTA	2′-O-methyl	20	6622
MOE	GCTAGCTAGCTAGCTAGCTA	2′-O-methoxyethyl	20	7657

**Table 3:** The oligonucleotide retention factor (*k*) and asymmetry factor (f_AS_) values were obtained for the dendrimeric stationary phase after three reaction cycles and various aqueous solutions of salts in mobile phases (without pH correction). Elution conditions: 0 min—0.125 M of salt, 5 min—0.4 M of salt, 15 min—0.4 M of salt.

Oligo	*k* ± SD							
	NaCl	f_AS_	NaClO_4_	f_AS_	CH_3_COONH_4_	f_AS_	HCOONH_4_	f_AS_
OL1	5.05 ± 0.04	1.1	1.35 ± 0.07	1.3	7.30 ± 0.09	1.7	8.53 ± 0.08	1.7
OL2	4.74 ± 0.00	0.9	1.13 ± 0.02	1.1	6.75 ± 0.05	1.5	8.25 ± 0.07	1.7
OL3	4.49 ± 0.06	0.9	0.68 ± 0.03	1.1	6.19 ± 0.02	1.6	7.09 ± 0.04	1.8
OL4	4.91 ± 0.02	1.0	1.25 ± 0.06	1.4	7.13 ± 0.02	1.6	6.72 ± 0.04	1.5
OL5	4.68 ± 0.04	1.2	0.57 ± 0.02	1.3	6.59 ± 0.02	1.7	6.38 ± 0.01	1.5
OL6	4.58 ± 0.01	1.1	0.69 ± 0.03	1.4	5.50 ± 0.07	1.5	6.56 ± 0.00	1.6
PS1	-		-		7.08 ± 0.02	1.7	6.75 ± 0.11	1.5
ME	5.82 ± 0.03	1.2	3.70 ± 0.02	1.2	6.62 ± 0.07	1.5	6.71 ± 0.09	1.6
MOE	4.87 ± 0.02	1.0	1.36 ± 0.03	1.2	6.35 ± 0.02	1.7	6.80 ± 0.15	1.7

-: not measured.

## Data Availability

Not applicable.

## Sample Availability

Samples of the compounds are available from the authors.
